# PVA:ALG Hybrid Bioink for Biofabrication of 3D Neural Models

**DOI:** 10.1021/acsomega.5c11570

**Published:** 2026-03-19

**Authors:** Lara Ece Celebi, Özüm Yildirim-Semerci, Ahu Arslan-Yildiz

**Affiliations:** † Department of Bioengineering, 52972 Izmir Institute of Technology (IZTECH), Izmir 35430, Turkey; ‡ Bioengineering Graduate Program, 6111 University of Notre Dame, Notre Dame, Indiana 46556, United States

## Abstract

Three-dimensional (3D) bioprinting technology has enabled the tunable and reproducible biofabrication of tissue models for in vitro neural tissue engineering. Combining natural and synthetic polymers offers a synergistic approach that harnesses the strengths of both materials to create bioinks with optimal printability and biocompatibility. In this study, a PVA/alginate hybrid bioink was developed for neural tissue engineering, and its suitability for bioprinting was evaluated through rheological analysis and pore factor characterization. Optimal bioprinting and cross-linking parameters were determined as 15% ALG, 16% PVA, 0.03 M GTA, and 5% CaCl_2_. Then, PVA/alginate scaffolds were characterized in terms of swelling and protein adsorption capacities, where ≥23-fold swelling and 1812.5 μg/mL protein adsorption capacities were reported. These findings show its potential to be utilized as a scaffold in neural tissue engineering. Neural cell proliferation, viability, and morphology were analyzed by culturing SH-SY5Y human neuroblastoma cells in 3D on hybrid scaffolds. Long-term cell viability was observed in 3D models through 15 days with a gradual increase, whereas in 2D cell culture, cell viability started to decrease after day 7 due to limitations of 2D cell culture. Moreover, increased extracellular matrix (ECM) secretion and neural marker expression of neural cells cultured on hybrid scaffolds were reported. 3D bioprinted PVA/Alginate scaffolds favored neural cell proliferation and have promise to be used in further neural tissue engineering applications, including modeling of neurodegenerative diseases in 3D and development of potential drugs.

## Graphical abstract

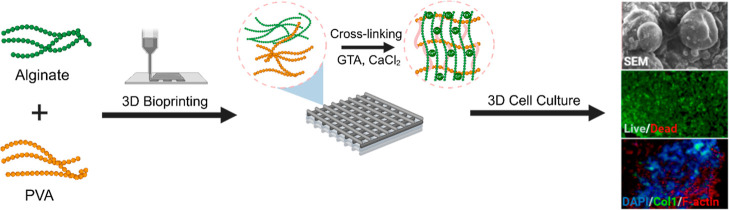

## 1 Introduction

Neurological disorders and neurodegenerative diseases have become prevalent in recent years.^1^ Various factors such as neuroinflammation,^2^ oxidative stress,^3^ protein misfolding,^4^ cytoskeletal abnormalities,^5,6^ and apoptosis^7^ could lead to neural degeneration, disrupting neural system and resulting in severe functional impairments.^8^ Tissue engineering has emerged as a promising approach to develop physiologically relevant in vitro neural culture platforms and biocompatible scaffolds that support neural cell growth. These platforms have the potential to enable controlled investigations of neurophysiology and to support in vitro evaluation of candidate therapeutic strategies.^9^

Over the years, various in vivo models^10,11^ have been utilized to study neuroscience. While animal models have been a valuable tool in neural research, they have limitations in terms of replicability, physiological differences, and ethical concerns.^12^ To overcome these obstacles, in vitro models were utilized, offering more controlled, realistic and reproducible experimental conditions, thereby presenting valuable tools for studying neurophysiology and neural regeneration.^13^ While two-dimensional (2D) cell cultures are simpler, cost-effective, and easier to handle, their limitations in replicating the physiological complexity of native tissues have led to the development and adoption of three-dimensional (3D) cell culture methods^14^ for neural tissue engineering approaches. Among these methods, 3D bioprinting is an emerging biofabrication method for producing scaffolds, which provides layer-by-layer deposition of bioink to create biomimetic tissue models^15^ by offering precise control, the ability to create complex 3D structures, maintained cell viability, personalized designs, and optimized material usage.^16,17^ Bioink is a specialized material that consists of cells and hydrogels and plays a pivotal role in the success of 3D bioprinting by influencing the viability and functionality of bioprinted tissues and constructs.^18^ Hydrogels and biopolymers are commonly utilized as bioink due to their biocompatibility, tunable viscosity, and high water retention capability.^19^ Natural hydrogels are known upon their biomimicry, and for promoting cellular interactions, making them ideal to create tissues that closely resemble native tissue.^20^ One of the commonly utilized natural hydrogels, Alginate (ALG), is a polysaccharide extracted from brown seaweed and often used in bioink formulations.^21–23^ However, it lacks the requisite stability when used in pristine form.^24^ Therefore, it is generally utilized in hybrid forms, or chemical modifications are required for optimal printability. In literature, ALG has been previously combined with synthetic polymers such as polylactic acid,^25^ poly­(ε-caprolactone) (PCL),^26^ and poly­(vinyl alcohol) (PVA)^27,28^ to enhance printability.^29^ PVA is a synthetic, biocompatible, and hydrophilic polymer that is commonly utilized as a supportive material in bioprinting applications.^30–32^ While earlier PVA–ALG systems mainly focused on material optimization and short-term evaluation using different cross-linking strategies and non-neural applications, here, alginate and PVA were utilized together to engineer a hybrid bioink for neural tissue engineering, as alginate provides biocompatibility, while PVA enhances mechanical stability, printability, and structural integrity without compromising cell viability. Notably, the developed physically and chemically cross-linked PVA/ALG network enables a balanced mechanical–biological performance, allowing high print fidelity and supporting long-term neural cell viability and neuronal marker expression.

In this study, a PVA/ALG hybrid bioink was employed for 3D bioprinting to be utilized in neural tissue engineering (Figure 1). Herein, suitability of the PVA/ALG hybrid bioink for 3D bioprinting was assessed via rheological analysis and pore factor characterization. Further, bioprinted PVA/ALG hybrid scaffolds were characterized in terms of swelling and protein adsorption capacities. Later, bioprinted scaffolds were evaluated for use in neural tissue engineering using SH-SY5Y neuroblastoma cell line by analyzing cell viability and extracellular matrix (ECM) formation. For the first time, this study reports the development of a PVA/ALG hybrid hydrogel as a promising bioink for 3D neural cell culture scaffolds.

**Figure 1:**
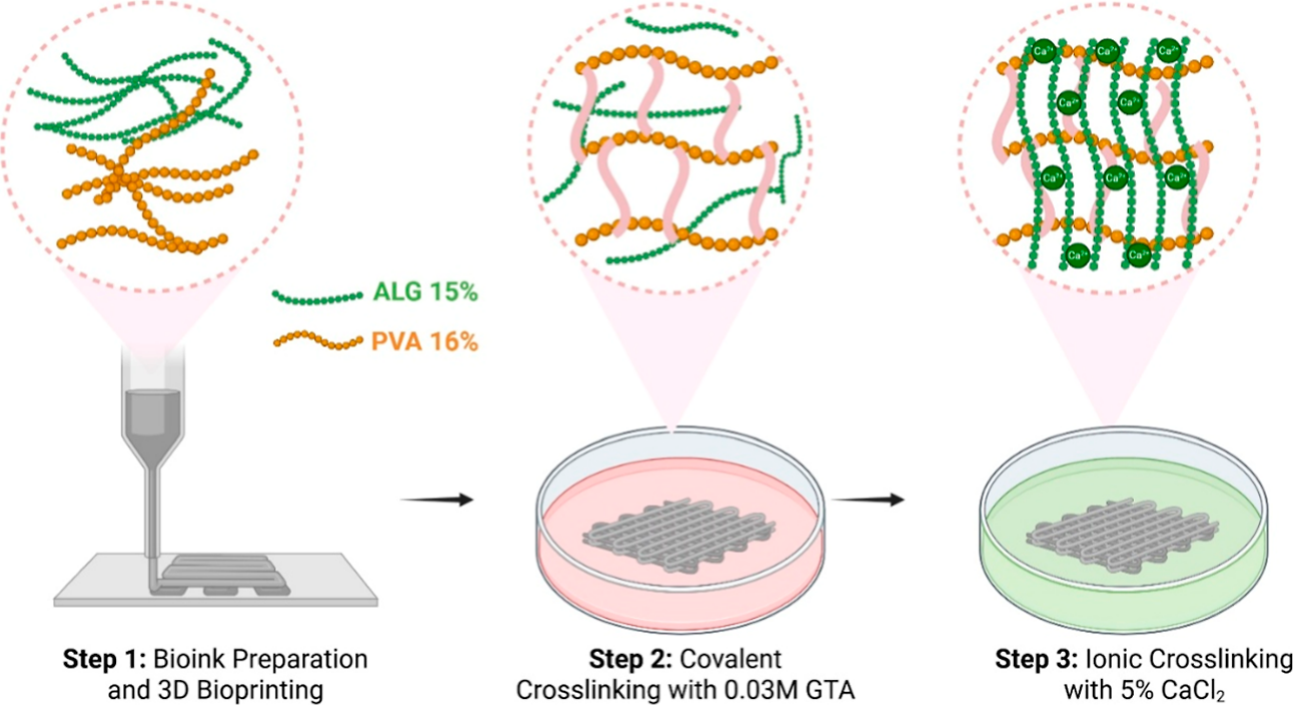
Schematic illustration of the bioink preparation and cross-linking process. **Step 1:** Preparation of ALG (15%) and PVA (16%) composite bioink and 3D bioprinting. **Step 2:** Covalent cross-linking of PVA in the printed construct using 0.03 M glutaraldehyde (GTA). **Step 3:** Ionic cross-linking of ALG with 5% CaCl_2_ to enhance the structural integrity and stability of the hydrogel network. (Created by authors by using Biorender).

## 2 Results and Discussion

Neural tissue engineering demands materials that combine chemical tunability with biological compatibility. Chemical properties enable precise control of bioink properties such as viscosity, cross-linking, and stability, while biological properties determine their capacity to support neuronal adhesion, ECM deposition, and neural maturation. Integrating these disciplines allows the creation of hybrid bioinks that are both bioprintable and capable of sustaining neural function.

### 2.1 Bioink Preparation and Printability Analysis

The successful preparation of the hybrid bioink was achieved by combining 16 wt % PVA and 15 wt % ALG hydrogels, which had been individually dissolved in ultrapure water at room temperature for 24 h. Mixing the two components in a 1:1 volume ratio resulted in a homogeneous blend, indicating good compatibility between polymers and the formation of a suitable matrix for subsequent applications. Then, cross-linking was done to obtain improved mechanical and physicochemical properties of bioprinted constructs.^33^ First, covalent cross-linking of bioprinted scaffolds was performed using 0.03 M GTA. Subsequently, 5% CaCl_2_ was used to cross-link the scaffold ionically, as it is a widely employed cross-linker for the physical cross-linking of ALG to form stiffer hydrogels (Figure 1).

Printability is a critical performance parameter in 3D bioprinting since it affects not only the 3D structure but also the mechanical and biological features of bioprinted constructs.^34^ Bioink viscosity and concentration are one of the most important parameters for printability.^35^ As shown in the printability chart (Figure 2A), most PVA–ALG combinations either exhibited free-flow behavior at low viscosities or resulted in irregular strand formation at higher ALG contents. Among all formulations, only 20% PVA:10% ALG and 16% PVA:15% ALG bioink were identified as the only compositions that provided stable extrusion with high print fidelity, without clogging or flow instability. In addition to its superior printability performance, the 16% PVA/15% ALG formulation was selected because it allows the bioink to be prepared using nearly equivalent polymer ratios, providing a balanced contribution from both components. Then, a viscosity analysis was performed to characterize the flow behavior of the bioinks. A rapid viscosity decrease with an increasing shear rate was observed for both the hybrid PVA/ALG and pristine ALG bioinks (Figure 2B), indicating pronounced shear-thinning behavior, which is essential for extrusion-based bioprinting as it enables reduced flow resistance under pressure while maintaining structural stability at low shear conditions.^36^ Power-law fitting of the shear stress–shear rate data (Figure 2C) quantitatively confirmed this behavior. The flow behavior indices (*n*) were calculated as 0.68 for pristine ALG and 0.77 for the PVA/ALG hybrid bioink, demonstrating clear non-Newtonian shear-thinning characteristics (*n* < 1).^37^ In contrast, pristine PVA exhibited near-Newtonian behavior (*n* ≈ 1.05), indicating minimal shear dependency.^38^ The consistency index (*K*) values further supported these observations, where pristine ALG showed a markedly higher *K* value (1458 Pa·s^*n*^), reflecting its highly viscous nature at low shear rates, while the PVA/ALG hybrid bioink displayed an intermediate K value (429 Pa·s^*n*^) (Table S1), suggesting a more balanced rheological profile.^39^ Pristine PVA, consistent with its low viscosity, exhibited the lowest *K* value (14.9 Pa·s^*n*^). These results indicate that blending PVA with ALG modulates viscosity while preserving shear-thinning behavior, yielding a rheological profile favorable for bioprinting applications.^40^

**Figure 2:**
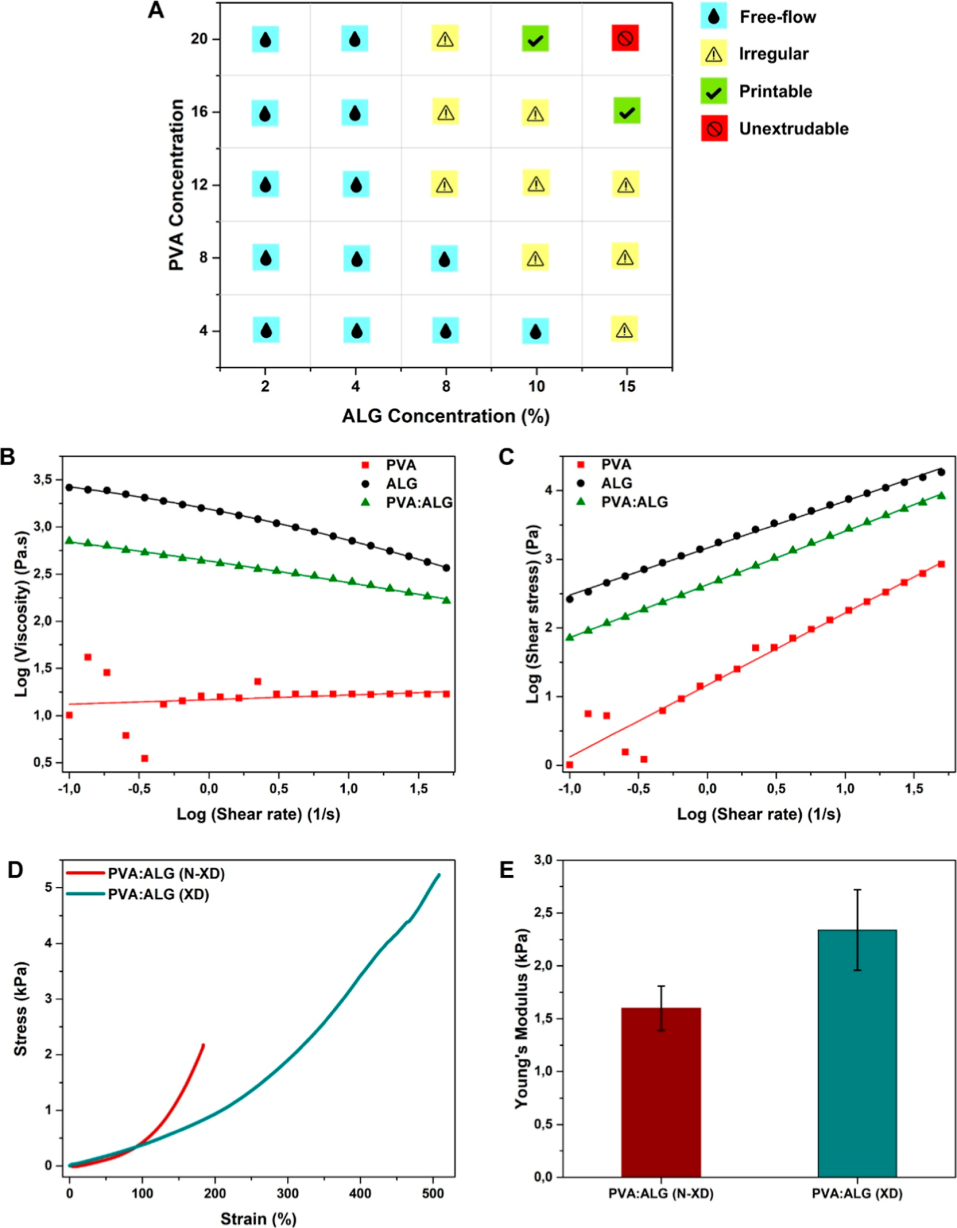
(A) Printability chart for PVA–ALG hybrid bioink across varying blend ratios, (B) viscosity vs shear rate plot, and (C) shear stress–shear rate plot of PVA, ALG, and PVA/ALG bioink. (D) Stress–strain curve obtained from the compression test and (E) Young’s modulus of PVA/ALG bioink before and after cross-linking.

Furthermore, mechanical analysis was done for the hybrid bioink before and after cross-linking process. Mechanical characterization results indicate a clear mechanical improvement following cross-linking of the PVA/ALG hybrid bioink (Figure 2D). The cross-linked (XD) samples withstood higher strains and stresses, while the non-cross-linked (N-XD) PVA/ALG exhibited earlier mechanical failure. Consistently, the Young’s modulus increased from 1.6 kPa (N-XD) to 2.34 kPa (XD) (Figure 2E), confirming enhanced stiffness due to network stabilization in hydrogel. Calculated Young’s modulus values fall within the soft mechanical range characteristic of neural tissues.^41–43^ These findings suggest that the developed bioink provides a mechanically favorable microenvironment for neural tissue engineering while maintaining sufficient structural stability for 3D applications.

For further printability studies, varied printing pressures were evaluated which is one of the most important parameters while optimizing printability.^44^ The pressure should be high enough to overcome the surface tension of bioink but low enough to maintain an ideal pore factor. Pore factor calculation was performed to quantitatively assess print fidelity by evaluating how closely the printed pores preserved the intended square geometry under different bioprinting pressures. To analyze the pore factor, the PVA/ALG bioink was bioprinted using a rectilinear grid model (Figure S1) at a pressure range of 3.8 and 5.4 psi (Figure 3A). As the pressure increased, square-shaped pores started to lose their regularity and print fidelity (Figure 3B). The calculated mean pore factor value was 1.067 at a pressure of 4.6 psi, which is the closest to the ideal value of 1 (Figure 3B). 16% PVA and 15% ALG (1:1 V/V) were determined as an optimum bioink formulation to be utilized in 3D bioprinting, based on the printability analysis results. Further characterizations were carried out using 16% PVA and 15% ALG, while a 25G nozzle and 4.6 psi pressure were utilized for bioprinting. It is noteworthy that the pristine forms of both bioinks were not printable under the applied conditions; hence, no data are reported for the individual bioinks.

**Figure 3:**
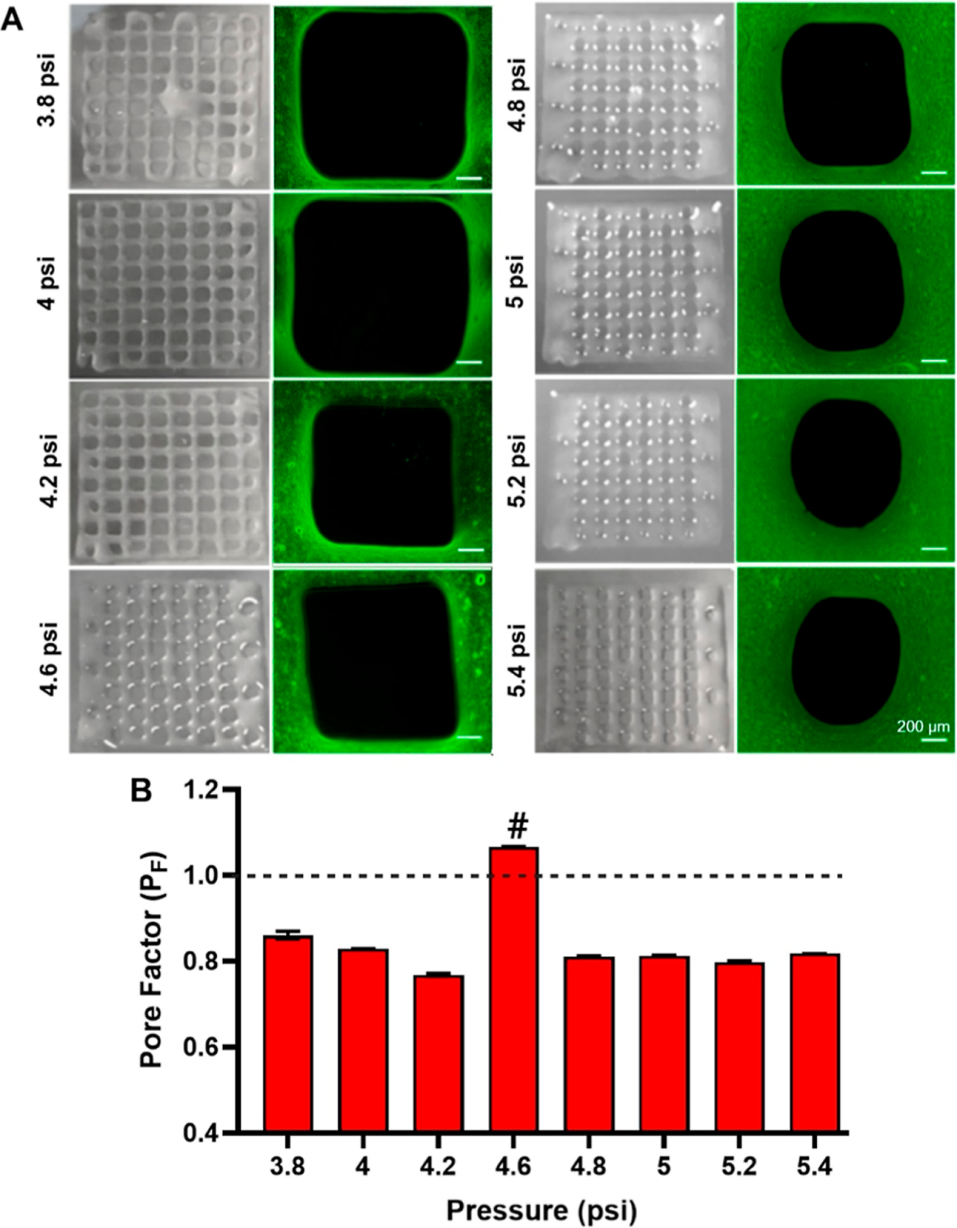
(A) Bioprinting of the rectilinear grid models (Scale bar: 200 μm). (B) Pore factor (P_F_) of bioprinted scaffolds with respect to different pressures. (Data are presented as mean ± standard error (*n* = 3). #A statistically significant difference was observed between the pore factor at 4.6 psi and those at the other applied pressures).

### 2.2 Characterization of Hybrid Scaffolds

Prior to the 3D cell culture studies, hybrid scaffolds were characterized through swelling and protein adsorption analyses. Swelling capacity of scaffolds is an important property to examine the volumetric changes in the aqueous environment and to gain information about their degradation characteristics.^45^ Swelling capacity of PVA/ALG scaffolds was recorded as ≈15-fold their own weight at 2 h and increased up to ≥23- fold at 24 h, and then, it reached equilibrium at the end of 48 h (Figure 4A). Findings demonstrated that the PVA/ALG scaffold exhibited an exceptional swelling capacity, significantly surpassing the 1000% threshold typically considered a high swelling ratio suitable for soft tissue engineering applications.^46^

**Figure 4:**
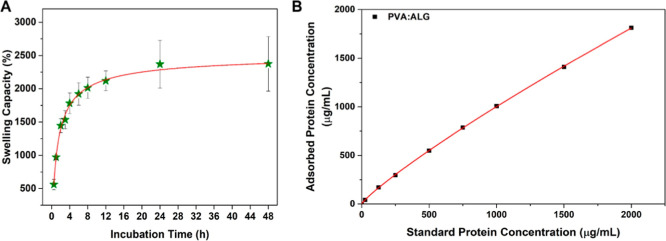
(A) Swelling capacity analysis of the hybrid scaffold. (B) Adsorbed protein concentrations fitting and error bars. Data are presented as mean ± SD (*n* = 3).

Protein adsorption capacity is directly related to surface properties and cell adhesion, hence it is important in providing insights into the adhesion of cells to scaffolds.^47^ The amount of adsorbed protein gradually increased and stabilized at different protein concentrations, indicating that the maximum protein adsorption was observed 1812.5 μg/mL for the highest standard BSA solution (Figure 4B). Results confirmed that PVA/ALG scaffolds effectively promoted protein adsorption, suggesting their potential to favor cell adhesion.

### 2.3 3D Neural Model Development

Formation of the 3D neural model was conducted by culturing SH-SY5Y cells for 15 days. Cell viability and proliferation are key indicators of biocompatibility, a scaffold’s ability to support neural tissue regeneration and long-term culture.^48^ Performance and suitability of the developed hybrid PVA/ALG scaffold in neural tissue engineering applications were evaluated by cell adhesion and viability analyses during long-term. Live/Dead analysis results showed that PVA/ALG scaffolds promoted formation of 3D cellular structures starting from day 5 (Figure 5A). Then, the cells covered the scaffold surface while maintaining high viability, indicating the hybrid scaffold supported sustained cell viability for 15 days. In contrast, high viability was observed in 2D control group initially, then cell death was seen after day 9, due to limitations in 2D cell culture, such as minimal surface area, contact-inhibition, and limited cell–matrix interaction.^49–51^ Complementary to Live/Dead assay results, Alamar Blue analysis revealed that cell viability in the hybrid PVA/ALG group gradually increased through 15 days and reached maximum cell viability compared to control groups (Figure 5B). In contrast, cell viability in 2D control increased until day 7, then it decreased in time, as expected. These findings suggest that 3D cell culture facilitates cell proliferation and enhances long-term cell survival, unlike 2D cell culture.

**Figure 5:**
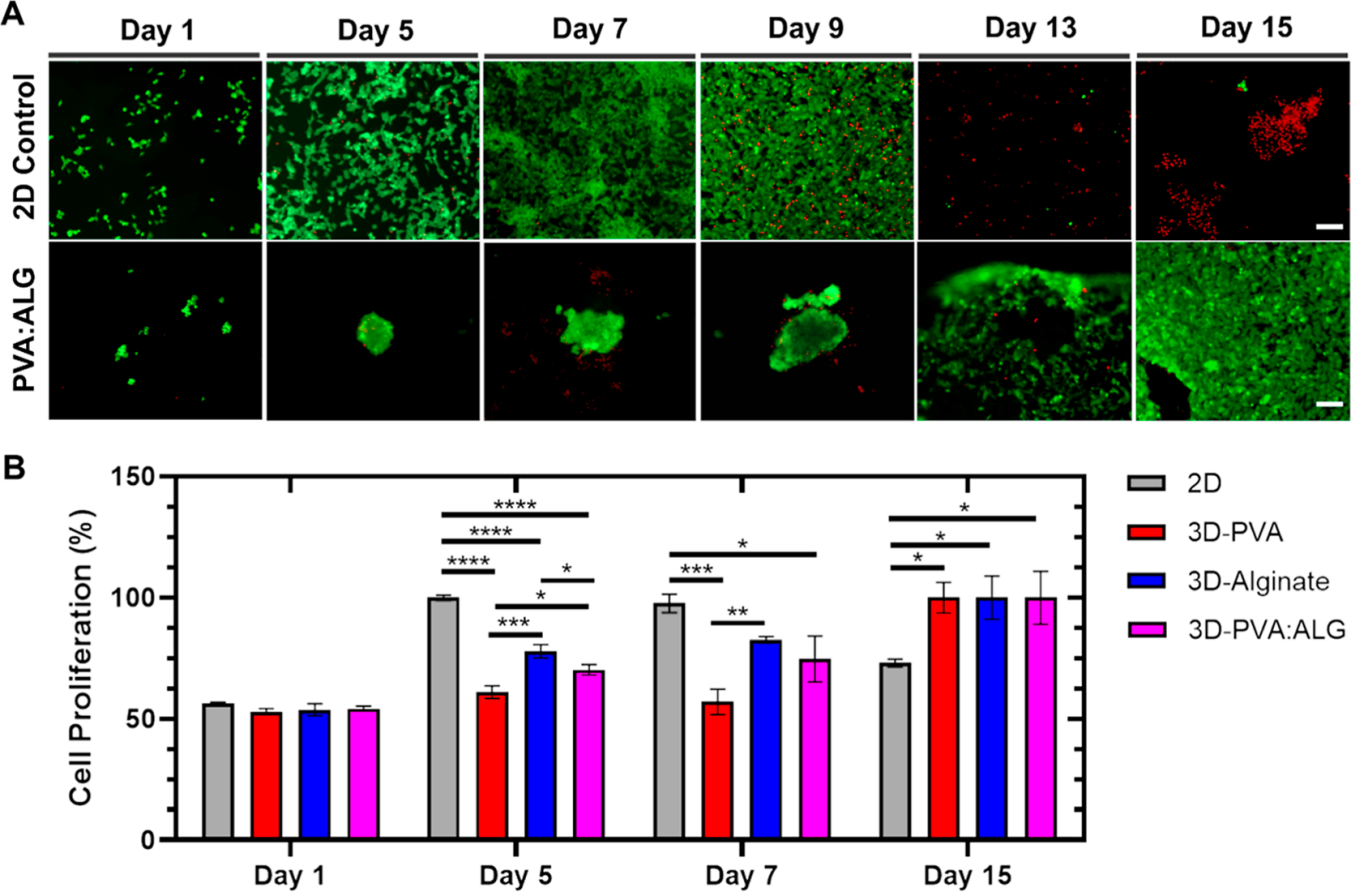
3D cell culture and viability analysis by (A) Live/Dead assay (Green: Live cell, Red: Dead cell) (scale bar: 100 μm) of SH-SY5Y cells cultured under 2D conditions and within 3D bioprinted hydrogel scaffolds. (B) Alamar Blue assay of SH-SY5Y cells cultured under 2D conditions and within 3D bioprinted hydrogel scaffolds (****p < 0.0001, ***p < 0.001, **p < 0.01, *p < 0.05). Data are presented as mean ± SD (*n* = 3).

Following the cell viability investigation, the morphology of SH-SY5Y cells on PVA/ALG hybrid scaffolds was monitored by SEM analysis for long-term. As shown in Figure 6, the SEM results revealed a progressive cell adhesion and proliferation through 15 days, supporting Live/Dead and Alamar Blue assay results. By day 7, an increased number of cells were observed with a more spread-out morphology, suggesting improved adhesion and proliferation compared to day 1 (Figure 6). By day 15, dense cell clusters homogeneously covered the scaffold surface, indicating favored cell proliferation and adhesion in 3D. Taken together, these findings demonstrate that the PVA/ALG hybrid scaffold provides a favorable 3D microenvironment for SH-SY5Y neuroblastoma cell adhesion, proliferation, and long-term viability, highlighting its high biocompatibility and suitability for neural tissue engineering applications.

**Figure 6:**
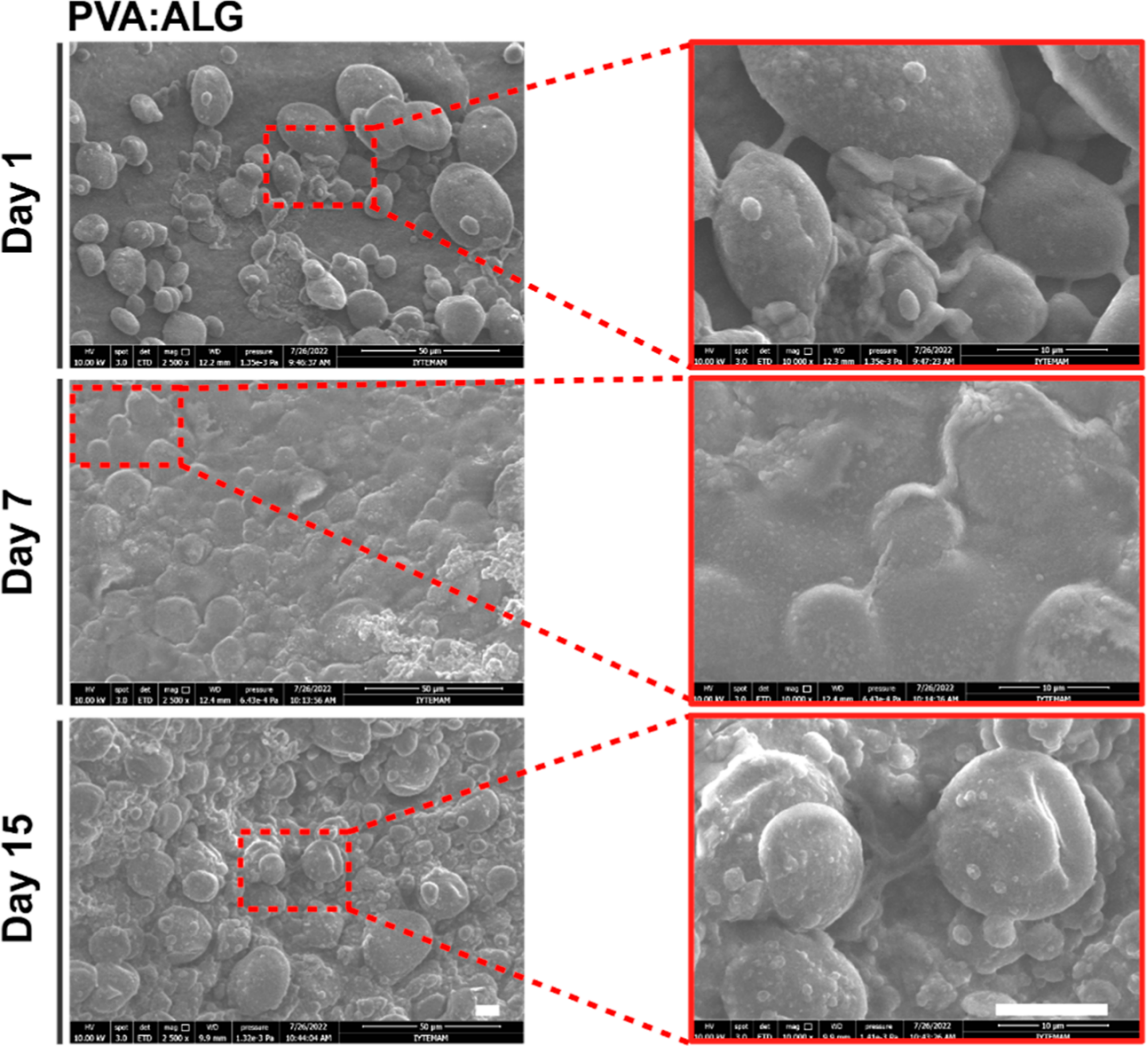
SEM Analysis of SH-SY5Y cells cultured on bioprinted hybrid scaffolds (scale bar: 10 μm).

### 2.4 Characterization of the 3D Neural Model by Immunostaining

Cellular and extracellular component secretion is a significant indicator of 3D model formation.^52^ Therefore, the analysis was conducted for the 3D neural model by immunostaining of F-actin and Collagen Type-I, along with nucleus staining using DAPI. Collagen is a major component of the ECM and provides structural support in tissues; hence, analyzing collagen secretion in 3D cultures helps the assessment of ECM organization.^53,54^ With Collagen Type-I immunostaining, ECM secretion of neural cells was validated on days 1, 7, and 15 (Figure 7). Collagen type-I secretion was observed as early as day 1, with a gradual increase at days 7 and 15 in the hybrid PVA/ALG scaffold (Figure 7A). While 2D culture exhibited a similar trend up to day 7, collagen expression declined by day 15, likely due to contact inhibition and cell death (Figure S2). Collagen deposition in 3D on day 15 exhibited a 13.3- and 1.7-fold increase compared to day 1 and day 7, suggesting enhanced matrix remodeling (Figure 7B). F-actin immunostaining is employed in assessing the cytoskeletal organization of cells within 3D cell cultures.^55^ Results showed that the cells cultured on 3D scaffolds appeared more interconnected and dispersed, suggesting enhanced cell–cell and cell–matrix interactions (Figure 7A). On the other hand, cells in 2D culture exhibited more defined and individual F-actin structures, a typical behavior of adherent monolayer cultures (Figure S2). Notably, a 12.95- and 1.03-fold increase in F-actin intensity was observed on day 15 in 3D culture compared to day 1 and day 7, indicating elevated actin secretion (Figure 7C). Taken together, the increased F-actin and collagen deposition in PVA/ALG scaffolds may be attributed to the 3D microenvironment providing multidirectional adhesion, which could enhance cell-ECM interaction, promoting actin secretion as well as ECM formation through upregulated collagen synthesis and deposition.^47^ Additionally, in 3D cell culture, the DAPI signal exhibited a continual increase over 15 days, whereas the DAPI signal in 2D culture correlated with the Live/Dead staining and Alamar Blue results, showing an increase in the cell number up to day 7, then followed by a decline due to limitations in 2D cell culture.^56,57^

**Figure 7:**
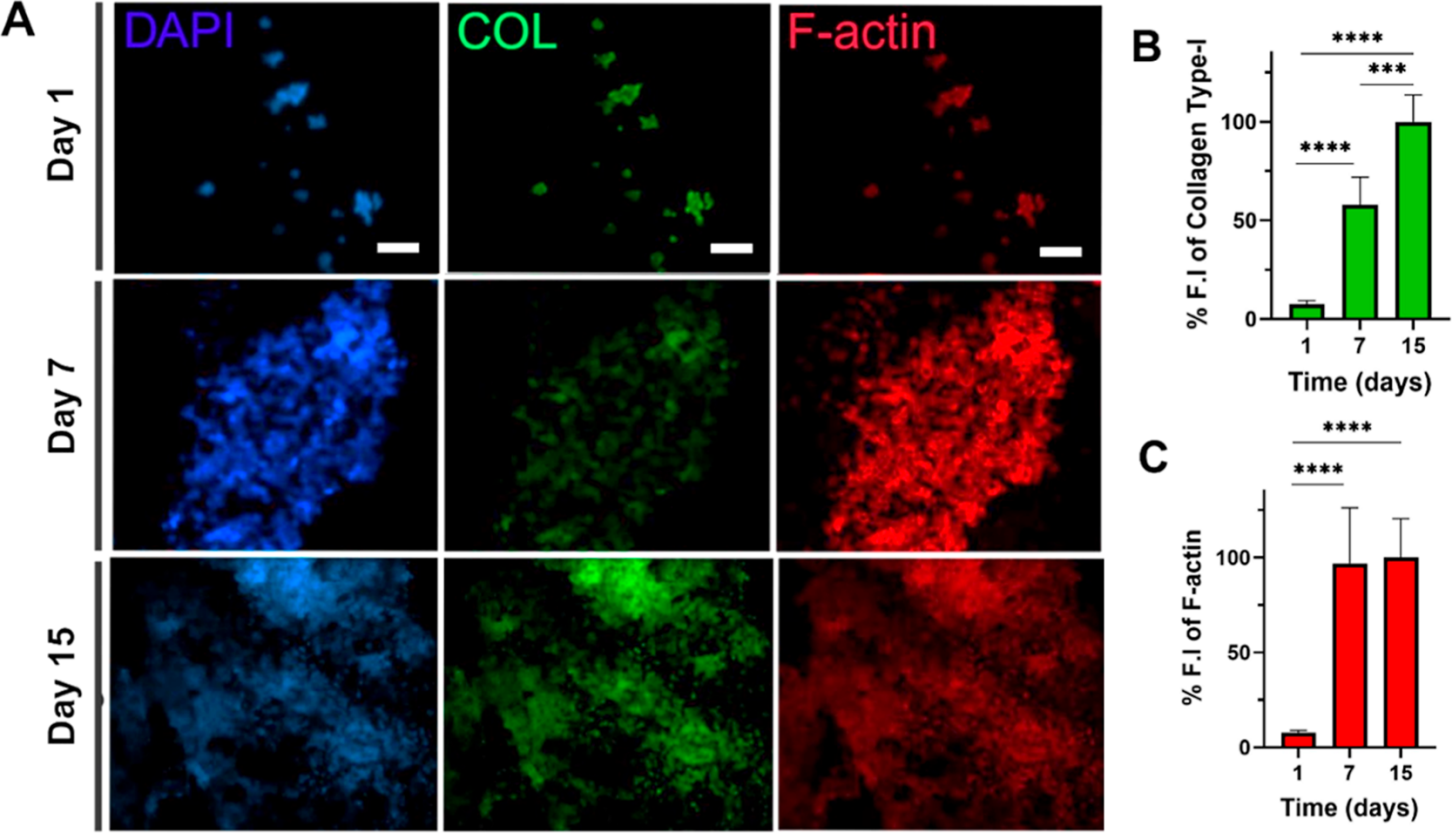
Cellular and extracellular components of cells in the 3D PVA/ALG scaffold. (A) Representative images show nuclei (DAPI, blue), collagen (COL, green), and F-actin (red) at days 1, 7, and 15 (scale bar: 100 μm). (B) Collagen type I fluorescence intensity (FI) at day 1, 7, and 15. (C) F-actin FI at day 1, 7, and 15. Data are presented as mean ± SD, ****p < 0.0001, ***p < 0.001.

In addition to cellular and extracellular component analyses, neural marker analysis was also done using NeuN, which is a mature postmitotic neuron marker^58^ to characterize neural cultures.^59^ According to the immunostaining results (Figure 8A), the majority of cells in the culture expressed NeuN, and F.I of NeuN in the 3D culture group was 2.01- and 1.06-fold higher on day 15 compared to days 1 and 7, respectively (Figure 8B). Increment in NeuN expression indicates that the developed 3D model is suitable for neuronal research, and developed hybrid bioink may promote neuronal maturation more effectively than traditional 2D cultures (Figure S3), potentially favoring neural development and function.^60,61^

**Figure 8:**
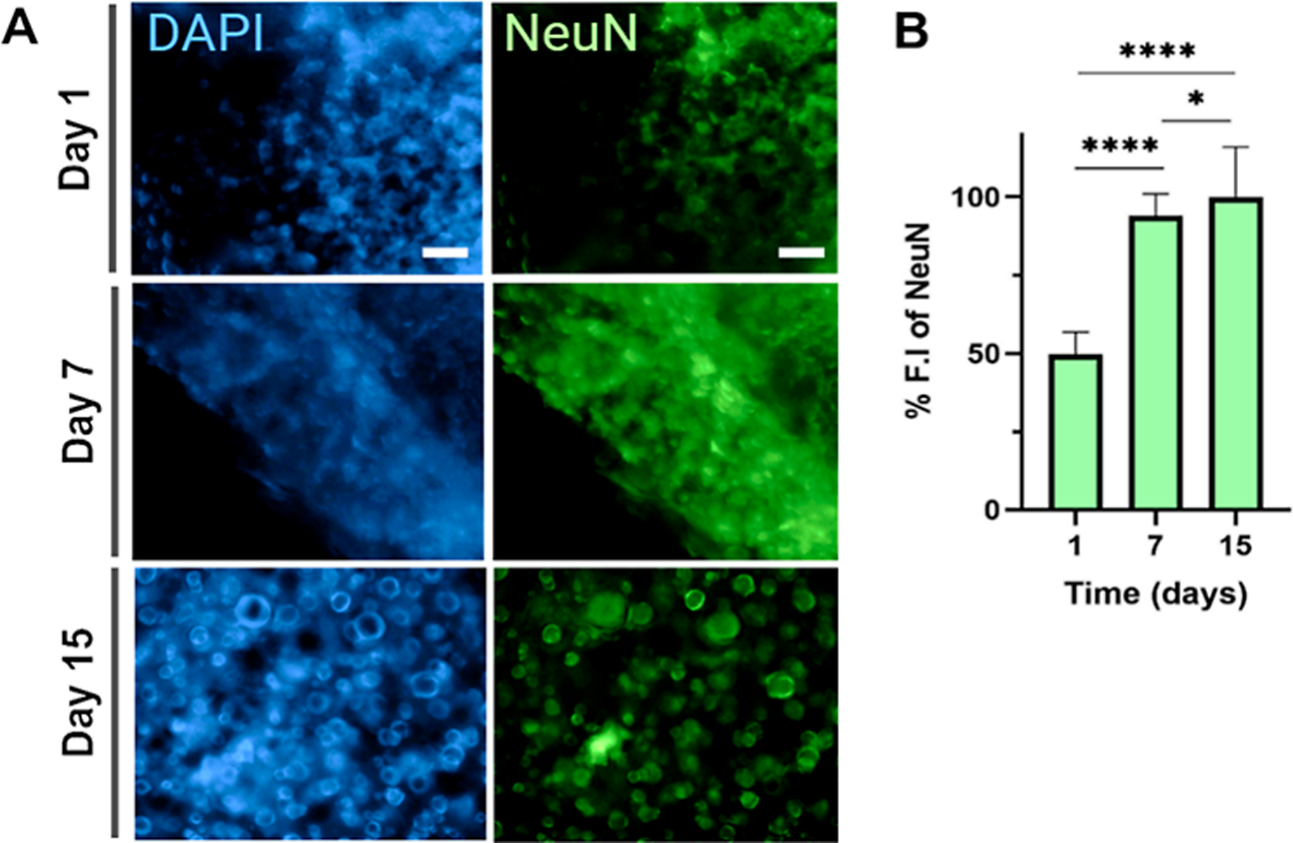
(A) Characterization of neuron-specific marker of SH-SY5Y cells cultured in the 3D PVA/ALG scaffold. Representative images show nuclei (DAPI, blue) and neuronal marker NeuN (green) at days 1, 7, and 15 (scale bar: 100 μm). (B) Quantification of NeuN F.I in SH-SY5Y cells cultured in 3D PVA/ALG scaffolds. NeuN expression at day 1, 7, and 15. Data are presented as mean ± SD, ****p < 0.0001, *p < 0.05.

## 3 Conclusion

In this study, a hybrid PVA/ALG bioink was successfully developed for 3D bioprinting, and its suitability for neural tissue engineering applications was demonstrated. Rheological analysis revealed a shear-thinning property, which is appropriate for 3D bioprinting. Pore factor analysis showed that the optimal pressure is 4.6 psi for bioprinting of 16 wt % PVA and 15 wt % ALG. Cross-linking with 0.03 M GTA and 5% CaCl_2_ resulted in structurally stable 3D scaffolds with favorable mechanical and physicochemical properties for 3D cell culture. Furthermore, characterization of the scaffolds confirmed high protein adsorption (1812.5 μg/mL) and swelling capacities (up to 23- fold at the end of 48 h), which showed the potential of developed scaffolds for cell adhesion and proliferation. Cell viability analyses using SH-SY5Y neuroblastoma cells demonstrated that hybrid scaffolds supported cell adhesion, 3D cellular structure formation, and sustained growth over 15 days, outperforming 2D cultures in maintaining long-term cell viability. Moreover, SEM imaging results confirmed progressive cell adhesion and proliferation, indicating that the scaffold provides a supportive 3D microenvironment for neural cell growth. Additionally, collagen and F-actin characterization highlighted the capability of the PVA/ALG scaffold to promote ECM deposition and cytoskeletal organization. Besides, NeuN immunostaining results showed high expression of neural markers in developed PVA/ALG scaffolds, indicating the potential of hybrid bioink for biofabrication of the 3D neural model via SH-SY5Y cells. Overall, the PVA/ALG hybrid scaffold presents a promising bioink for neural tissue engineering by providing structural integrity, biocompatibility, and a favored 3D microenvironment for neuronal cell growth. Future iterations of the model can incorporate neuronal differentiation and functional readouts such as neurite outgrowth and branching, as well as disease or drug response evaluations to characterize the utility of this platform for disease modeling and therapeutic screening.

## 4 Experimental Section

### 4.1 Materials

3D-Bioprinter (AxolotlBio Systems) was utilized for bioprinting cell-laden constructs. Poly­(vinyl alcohol) (PVA, wt 30,000–70,000), sodium alginate (A1112, low viscosity, viscosity: 4–12 cP), calcium chloride (CaCl_2_), and glutaraldehyde (GTA) were purchased from Sigma-Aldrich. Ethanol (99%), acetone, and hydrochloric acid (HCl) (37%) were purchased from Isolab. Fluorescein (Fluka Analytical) was used to visualize the bioprinted constructs by using fluorescence microscopy (Zeiss Axio Observer). Lyophilized bovine serum albumin powder (BSA A9418, Sigma-Aldrich), bicinchoninic acid (BCA) assay (Pierce, Thermo Scientific), and phosphate buffer saline (PBS, pH 7.4 70011-044, Gibco-Thermo Fischer) were utilized for protein adsorption analysis. SH-SY5Y neuroblastoma cell line (ATCC CRL-2266) was utilized in cell culture studies. Cell media were prepared using high glucose Dulbecco’s modified Eagle’s medium (DMEM, 41965-039, Gibco), 15% (v/v) fetal bovine serum (FBS, 10270-106, Gibco), and 1% penicillin–streptomycin (P/S, P4333, Sigma-Aldrich). Trypsin–EDTA solution (25200-056, Gibco), dimethyl sulfoxide (DMSO) (99,9%, Carlo Erba), and Trypan Blue (Sigma-Aldrich, USA) were used. Live/Dead cell viability tests were conducted using CytoCalcein Green and Propidium Iodide (PI) (AAT Bioquest) dyes. For immunostaining, Triton X-100 (0.1%, Amresco, OH, US), BSA (1%), F-Actin labeled with TRITC-conjugated Phalloidin (Sigma-Aldrich), anticollagen Type I labeled with FITC (Sigma-Aldrich), and DAPI (Sigma-Aldrich) were purchased. Expression of neurospecific marker was validated using anti-NeuN (Abclonal, A19086). Alamar Blue was purchased from Santa Cruz Biotechnology Inc. (USA) and the absorbance values were obtained using a microplate reader (Fisher Scientific accuSkan GO UV/Vis Spectrophotometer). Lastly, a scanning electron microscope (FEI QUANTA, 250 FEG) was used for SEM measurements.

### 4.2 Methods

#### 4.2.1 Hybrid Bioink Preparation and Printability Analysis

Hybrid bioink preparation was done by dissolving 16 wt % PVA and 15 wt % alginate (ALG) hydrogels in ultrapure water for 24 h at RT and mixing them in a 1:1 volume ratio. Bioprinting studies were conducted by employing the Axo Bioprinting System (Axolotl Biosystems Ltd., Turkey), where 3D design and slicing were done using SolidWorks and Repetier Host software, respectively. A rectilinear grid model was used to optimize bioprinting parameters through printability analysis of a hybrid bioink. Bioprinting studies were carried out using following parameters: 0.5 and 8.0 psi pressure, 25G nozzle diameter, 10 mm/s printing speed, and 50% infill density (Table S2). Pristine PVA and ALG bioinks were used as control groups.

For printability analysis, 2.5% Fluorescein was added to developed bioink and visualized under a fluorescence microscope. Pore factor (P_F_) was determined using established equations,^18^ quantitative analysis was conducted with ImageJ software, and obtained data were plotted using GraphPad Prism. Rheological analysis of the PVA, ALG, and hybrid bioink was performed to evaluate the shear thinning behavior using a rheometer (Anton Paar, MCR-102e) within a 0:50 s^–1^ shear rate at 25 °C.

Prior to 3D cell culture studies, hybrid scaffolds were both chemically and physically cross-linked using GTA and CaCl_2_. All experiments involving glutaraldehyde were conducted in a fume hood with appropriate personal protective equipment due to its toxic properties. First, PVA/ALG scaffolds were exposed to 0.03 M GTA solution for 30 min. Following GTA cross-linking, PVA/ALG hybrid scaffolds were thoroughly rinsed with ethanol and subsequently washed multiple times with ultrapure water to remove unreacted GTA residues before CaCl_2_ treatment. Then, they were immersed in a 5% CaCl_2_ solution for 15 min. After ionic cross-linking with CaCl_2_, an additional washing step with ultrapure water was performed to eliminate excess Ca^2+^ ions. Besides, pristine PVA scaffolds were chemically cross-linked in 0.03 M GTA solution for 30 min followed by rinsing with ethanol and ultrapure water to remove GTA residues. Pristine ALG scaffolds were ionically cross-linked via incubation in a 5% CaCl_2_ solution for 15 min followed by rinsing with ultrapure water. The mechanical properties of the PVA/ALG bioink before and after cross-linking were evaluated using a Texture Analyzer (Stable Micro Systems, TA.XT Plus C). A compression test was carried out with a 5 kg load cell and a 35 mm diameter probe at a compression rate of 0.5 mm/s. Young’s modulus (E) was calculated from the linear region of the stress–strain profiles.^62^

#### 4.2.2 Characterization of Hybrid Scaffolds

Prior to 3D cell culture studies, PVA, ALG, and PVA/ALG hybrid scaffolds were characterized through the determination of swelling and protein adsorption capacities. For this, scaffolds were bioprinted by using parameters optimized through printability analysis. After that, swelling capacity of 3D bioprinted scaffolds was assessed at specific time points (*t* = 0, 0.5, 1, 2, 3, 4, 6, 8, 24, 48 h) by comparing the wet and dry mass before and after PBS immersion as described elsewhere.^62^ Swelling capacity was calculated gravimetrically using the formula ((*W*_t_–*W*_d_)/*W*_d_) × 100, where *W*_d_ represents the dry weight and W_t_ represents the wet weight of the scaffolds, and then, data were plotted using GraphPad Prism software (GraphPad Prism, Inc., San Diego, USA). Moreover, the protein adsorption capacity of scaffolds was analyzed using a BCA assay (Pierce, Thermo Scientific). Hybrid scaffolds were immersed in 0–2000 μg mL^–1^ BCA solution and incubated at 37 °C for 2 h. Finally, absorbance values were measured at 562 nm using a UV/Vis microplate spectrophotometer (Fisher Scientific accuSkan GO), and the results were plotted by using GraphPad Prism software.

#### 4.2.3 3D Neural Model Development and Characterization

A 3D neural tissue model was developed using the SH-SY5Y human neuroblastoma cell line. Standard 2D culture of cells was maintained in DMEM media containing 15% FBS and 1% P/S, and then, cells were detached using trypsin–EDTA when they reached 80–90% confluency. 35 × 10^3^ cells were seeded on each UV-sterilized PVA, Alginate, and hybrid scaffold, and cultured through 15 days. Cell viability was analyzed using the Live/Dead assay, where CytoCalcein Green and PI dyes were utilized to visualize live and dead cells, respectively. Then, the cells were observed under a fluorescence microscope. Besides, the Alamar Blue assay was done to assess cell viability quantitatively, where the cells were incubated with 0.01% resazurin sodium salt for 4 h and measured using a spectrophotometer at 570–600 nm. Cell morphology on the PVA/ALG hybrid scaffold was analyzed by SEM on days 1, 7, and 15, where the cells were fixated using 4% paraformaldehyde (PFA).

Cellular and extracellular components were analyzed by immunostaining of nucleus, cytoskeleton, and Collagen type-I. Cells were fixated in 4% PFA on days 1, 7, and 15, and they were permeabilized and blocked as described elsewhere.^18^ Actin cytoskeletons and Collagen Type-I secretion were visualized by immunostaining with TRITC-conjugated Phalloidin and anti-Collagen Type I, and then, DAPI staining was done for nucleus monitoring. Furthermore, expression of neuron-specific marker was validated using anti-NeuN and Alexa Fluor 488 conjugated anti-Rabbit IgG, then visualized by a fluorescence microscope. Fluorescence intensity (FI) analysis for F-actin, Collagen Type-I, and NeuN signals was performed by using ImageJ/Fiji (NIH) software. Background subtraction was uniformly applied to all images. Positive fluorescence regions were identified via an identical thresholding procedure, converted to binary masks, and saved as ROIs. Quantification was conducted by measuring the mean gray value of each ROI. FI values were normalized and expressed as relative percentages.

#### 4.2.4 Statistical Analysis

Statistical analysis was conducted through one-way analysis of variance (ANOVA) followed by Tukey’s post hoc test to identify significant differences. For comparisons between two groups, Student’s *t*-test was applied. All *p*-values were two-sided, with *p* < 0.05 being statistically significant. Each experiment was performed with a sample size (*n*) of at least 3.

## Supplementary Material



## Notes

Design and 3D rendering of the printed scaffold; fluorescence-based characterization of cellular, extracellular, and neuron-specific markers in SH-SY5Y cells under 2D control conditions across days 1, 7, and 15; power-law fitting parameters (*n*, *K*, *R* ^2^) derived from rheological analysis; and bioprinting parameters used for scaffold fabrication (PDF)

## Notes

Bioengineering Graduate Program, University of Notre Dame, Notre Dame, IN 46556, USA Email: lcelebi@nd.edu

## Abbreviations

3D: three-dimensional
2D: two-dimensional
ALG: alginate
BCA: bicinchoninic acid
BSA: bovine serum albumin
DAPI: 4′,6-diamidino-2-phenylindole
DMEM: Dulbecco’s modified Eagle’s medium
DMSO: dimethyl sulfoxide
ECM: extracellular matrix
FBS: fetal bovine serum
F.I.: fluorescence intensity
GTA: glutaraldehyde
PFA: paraformaldehyde
PBS: phosphate-buffered saline
PI: propidium iodide
PVA: poly­(vinyl alcohol)
SEM: scanning electron microscopy
