# Supplementary material for: PVA:ALG Hybrid Bioink for Biofabrication of 3D Neural Models
Source: ACS Omega. 2026 Mar 19;11(12):18999–9009. doi: 10.1021/acsomega.5c11570 (PMC13044844; doi:10.1021/acsomega.5c11570)
Supplement: Supplementary file 1 [file ao5c11570_si_002.pdf]

PVA:ALG Hybrid Bioink for Biofabrication of 3D Neural Model

Lara Ece Celebi[1,2], Özüm Yildirim-Semerci[1*], Ahu Arslan-Yildiz[1*]

[1]*Department of Bioengineering, Izmir Institute of Technology (IZTECH), 35430 Izmir, Turkey*

[2]*Present address: Bioengineering Graduate Program, University of Notre Dame, Notre Dame, IN 46556, USA*

**Co-corresponding authors: ahuarslan@iyte.edu.tr; ozumyildirim@iyte.edu.tr*

Supplementary Table 1. Summary of power-law fitting results for the investigated bioink formulations. The flow behavior index (n), consistency index (K), and coefficient of determination (R^2) were calculated by fitting the experimental shear stress–shear rate data to the power-law model ($\tau = K \cdot \gamma^n$) over the shear rate range of 0–50 s^{-1}.

	PVA	***ALG***	***PVA:ALG***
K	14,9	1458	429
n	1,05	0,68	0,77
R²	0,95	0,99	0,99

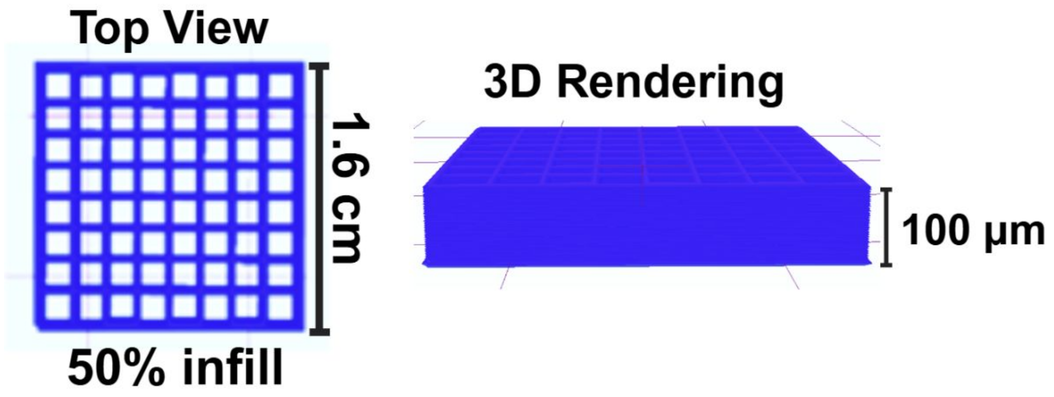

Supplementary Figure 1. Design of the 3D-printed scaffold. Top view shows a 1.6 cm square scaffold with 50% infill pattern. The 3D rendering shows the final construct with a thickness of 100 µm.

Supplementary Table 2. Bioprinting parameters for fabrication of the hybrid PVA–ALG rectilinear grid constructs.

Model	C_{PVA} (%)	C_{ALG} (%)	Nozzle Size	Speed	Applied Pressure Range	Optimized Pressure	Infill Density	Layer Height
Rectilinear grid	16%	15%	25G (250μm)	10 mm/s	0.5-8.0 psi	4.6 psi	50%	100μm

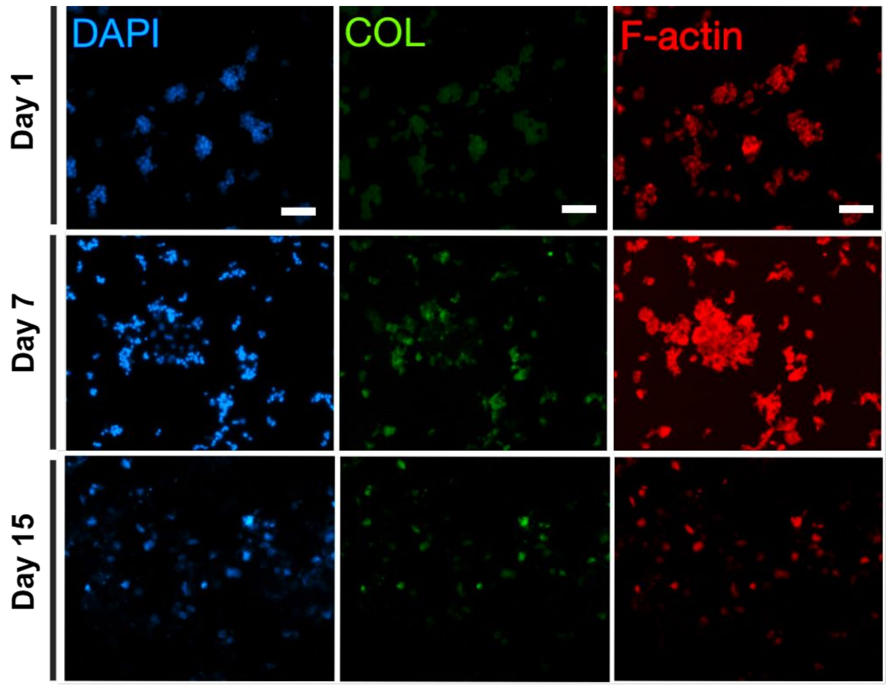

Supplementary Figure 2. Cellular and extracellular components of cells in 2D control. Representative images show nuclei (DAPI, blue), collagen (COL, green), and F-actin (red) at days 1, 7, and 15 *(scale bar: 100 μm)*.

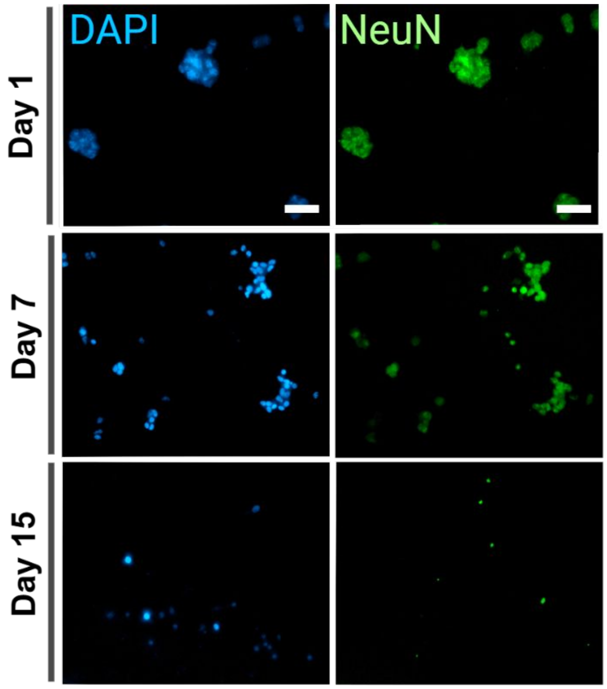

Supplementary Figure 3. Characterization of Neuron-specific Marker of SH-SY5Y cells cultured in 2D control. Representative images show nuclei (DAPI, blue) and neuronal marker NeuN (green) at days 1, 7, and 15 *(scale bar: 100 µm)*.
